# 11β-Hydroxysteroid Dehydrogenase 1 Specific Inhibitor Increased Dermal Collagen Content and Promotes Fibroblast Proliferation

**DOI:** 10.1371/journal.pone.0093051

**Published:** 2014-03-25

**Authors:** Mika Terao, Mamori Tani, Saori Itoi, Takuji Yoshimura, Toshimitsu Hamasaki, Hiroyuki Murota, Ichiro Katayama

**Affiliations:** 1 Department of Dermatology, Graduate School of Medicine, Osaka University, Suita, Osaka, Japan; 2 Laboratory of Reproductive Engineering, The Institute of Experimental Animal Sciences, Osaka University Medical School, Suita, Osaka, Japan; 3 Department of Biomedical Statistics, Graduate School of Medicine, Osaka University, Suita, Osaka, Japan; University of Ulm, Germany

## Abstract

Glucocorticoids (GCs) are one of the most effective anti-inflammatory drugs for treating acute and chronic inflammatory diseases. However, several studies have shown that GCs alter collagen metabolism in the skin and induce skin atrophy. Cortisol is the endogenous GC that is released in response to various stressors. Over the last decade, extraadrenal cortisol production in various tissues has been reported. Skin also synthesizes cortisol through a *de novo* pathway and through an activating enzyme. 11β-hydroxysteroid dehydrogenase 1 (11β-HSD1) is the enzyme that catalyzes the conversion of hormonally inactive cortisone to active cortisol in cells. We previously found that 11β-HSD1 negatively regulates proliferation of keratinocytes. To determine the function of 11β-HSD1 in dermal fibroblasts and collagen metabolism, the effect of a selective 11β-HSD1 inhibitor was studied in mouse tissues and dermal fibroblasts. The expression of 11β-HSD1 increased with age in mouse skin. Subcutaneous injection of a selective 11β-HSD1 inhibitor increased dermal thickness and collagen content in the mouse skin. *In vitro*, proliferation of dermal fibroblasts derived from 11β-HSD1 null mice (*Hsd11b1^−/−^* mice) was significantly increased compared with fibroblasts from wild-type mice. However, *in vivo*, dermal thickness of *Hsd11b1^−/−^* mice was not altered in 3-month-old and 1-year-old mouse skin compared with wild-type mouse skin. These *in vivo* findings suggest the presence of compensatory mechanisms in *Hsd11b1^−/−^* mice. Our findings suggest that 11β-HSD1 inhibition may reverse the decreased collagen content observed in intrinsically and extrinsically aged skin and in skin atrophy that is induced by GC treatment.

## Introduction

Glucocorticoids (GCs) have been used for more than half a century as one of the most effective anti-inflammatory drugs for treating acute and chronic inflammatory diseases. Topically applied GCs are one of the most common ointments used by dermatologists to treat inflammatory dermatitis. The mechanism of GCs is thought to be an anti-inflammatory effect on keratinocytes and on skin-infiltrating inflammatory cells. Skin atrophy is a known side effect of both topical and systemic GC treatment[1], [2]. Several studies show that GCs alter collagen metabolism in the skin [3], [4], [5].

Cortisol is the endogenous GC in humans (corticosterone in rodents) that is released in response to various stressors such as physical injury and psychological stress. It regulates multiple biological processes, including growth, development, metabolism, and behavior [6], [7]. Endogenous GC is also reported to play a role in suppressing disease activity, such as that of rheumatoid arthritis [8], [9].

Over the last decade, research has focused on not only systemic cortisol/corticosterone production by the hypothalamic-pituitary-adrenal axis, but also local cortisol/corticosterone production by *de novo* synthesis and by an activating enzyme [10]. Extraadrenal production of cortisol/corticosterone has been reported in tissues such as the colon [11], [12], [13], heart [14], [15], [16], and lung [17]. Recently, local cortisol/corticosterone production in the skin via a *de novo* pathway and via the cortisol activating enzyme, 11β-hydroxysteroid dehydrogenase (11β-HSD) has been reported [18], [19], [20], [21], [22], [23], [24], [25], [26], [27].

11β-HSD catalyzes the interconversion between hormonally active cortisol/corticosterone and inactive cortisone/11-dehydrocorticosterone (11-DHC) in cells [28], [29], [30]. The two isoenzymes of 11β-HSD both reside in the membrane of the endoplasmic reticulum [31]. The 11β-HSD1 isoform, which catalyzes the conversion of cortisone/11-DHC to cortisol/corticosterone, is widely expressed and present at the highest levels in the liver, lungs, adipose tissues, ovaries, and central nervous system. The 11β-HSD2 isoform, which catalyzes the conversion of cortisol/corticosterone to cortisone/11-DHC, is highly expressed in distal nephrons, the colon, sweat glands, and the placenta. In a previous study, we found that 11β-HSD1 negatively regulates the proliferation of keratinocytes both *in vivo* and *in vitro* and proliferation of fibroblasts *in vivo*. Applying a selective inhibitor of 11β-HSD1 to the dorsal skin of mice increases the number of cells positive for the Ki-67 antigen and results in epidermal thickening and enhanced wound healing [22].

In the current study, we investigated the role of 11β-HSD1 in dermal fibroblasts. Our results demonstrate that inhibition of 11β-HSD1 modulates collagen metabolism by increasing the number of dermal fibroblasts. Because the expression of 11β-HSD1 increased with age in the mouse skin, 11β-HSD1 could be a target for treating dermal atrophy induced by aging or GC treatment.

## Materials and Methods

### Ethics statement

Animal care was in strict accordance with the institutional guidelines of Osaka University. All of the animal experiments were carried out with the approval of the Animal Experiments Committee of Osaka University (#20-003-0).

### Cell culture

Isolation and culture of mouse fibroblasts were carried out as previously described [32]. Full-thickness skin harvested from 2- to 4-day-old newborn mice was treated with 4 mg/ml dispase (Gibco; Invitrogen, Paisley, UK) for 1 h at 37°C. Next, the epidermis was peeled from the dermis, which was placed in phosphate-buffered saline (PBS) +0.05% type-1 collagenase (Sigma-Aldrich, St. Louis, MO, USA) and incubated at 37°C for 30 min with vigorous agitation to prepare single cells. After filtration, cells were centrifuged at 200×*g* for 10 min, resuspended in DMEM +10% FBS, and incubated at 37°C in 5% CO_2_. First or second passage fibroblasts were used for experiments.

### Mice

C57BL/6 mice were obtained from Clea Japan Ltd. (Osaka, Japan), and Hos: HR-1 mice were obtained from SLC Japan Ltd. (Tokyo, Japan).

### Generation of the *Hsd11b1* null mouse (*Hsd11b1^−/−^* mouse)

*Hsd11b1 ^tm1a/+^* ES cells were purchased from the Knockout Mouse Project (KOMP) Repository (Davis, CA, USA). The ES cells were injected into blastocysts collected from superovulated BALB/c female mice. The treated blastocysts were then transferred into the uterus of pseudopregnant ICR female mice (SLC Japan) to obtain chimeric mice. Male chimeras were mated with female C57BL/6 mice, resulting in germline transmission of the *Hsd11b1 tm1a* allele. Mouse genotyping was performed with PCR using the following primers: 5′-catacacattgcccttgtgc-3′ and 5′-ccctcaaggccagattggtatat-3′ for the wild-type allele; 5′-gaaagtataggaacttcgtcg-3′ and 5′-ccctcaaggccagattggtatat-3′ for the *Hsd11b1 tm1a* allele. *Hsd11b1* ^tm1a/tm1a^ mice were obtained by intercrossing *Hsd11b1^ tm1a/+^* mice. The *Hsd11b1 tm1a* allele is regarded as a null allele, because in *Hsd11b1 ^tm1a/tm1a^* mice, no expression was detected at either the mRNA or protein levels.

### Histopathological analysis

Samples were fixed in 10% formaldehyde for 24 h, followed by embedding in paraffin and microtome sectioning. Sections (4 μm thick) were stained with hematoxylin and eosin (H&E). The amount of collagen was evaluated after tissue staining with the Masson's trichrome technique.

### Western blotting

Skin samples were crushed in liquid nitrogen and solubilized at 4°C in lysis buffer (0.5% sodium deoxycholate, 1% Nonidet P40, 0.1% sodium dodecyl sulphate, 100 μg/ml phenylmethylsulphonyl fluoride, 1 mM sodium orthovanadate, and protease inhibitor cocktail). Thirty micrograms of protein were separated on SDS-polyacrylamide gels and transferred onto polyvinylidine fluoride membranes (Bio-Rad, Hercules, CA, USA). Non-specific protein binding was blocked by incubating the membranes in 5% w/v non-fat milk powder in TBS-T (50 mM Tris-HCl, pH 7.6, 150 mM NaCl, and 0.1% v/v Tween-20). The membranes were incubated with rabbit anti-collagen type1 antibody (Merck, Whitehouse Station, NJ, USA) or sheep anti-11β-HSD1 antibody (The Binding Site, Birmingham, UK) diluted 1∶1000 overnight at 4°C or with mouse anti-β-actin antibody (Sigma-Aldrich) diluted 1∶10,000 for 30 min at room temperature. Then, the membranes were washed three times in TBS-T for 5 min. Finally, the membranes were incubated with either HRP-conjugated anti-mouse, anti-rabbit, or anti-sheep antibody at a dilution of 1∶10,000 for 60 min at room temperature. Protein bands were detected using the ECL Plus kit (GE Healthcare, Buckinghamshire, UK). The intensity of the bands was quantified using NIH image J software.

### Selective 11β-HSD1 inhibitor

The 11β-HSD1 inhibitor, 385581 (Merck, Darmstadt, Germany), is a potent inhibitor of 11β-HSD1 with >450- and >100-fold selectivity over human and mouse 11β-HSD2, respectively [33]. The inhibitor was dissolved in DMSO and further diluted more than 100,000-fold in culture medium (for *in vitro* experiments) or in PBS (for *in vivo* experiments). DMSO was used as a vehicle control.

### 11β-HSD1 inhibitor treatment *in vivo*

The dorsal skin of 7-week-old and 1-year-old C57BL/6 mice (N = 6) was subcutaneously injected with the 11β-HSD1 inhibitor (10 μM, right side) or DMSO (left side) dissolved in PBS once a day for 21 days. One day after the last injection, the dorsal skin was harvested for histological analysis.

### MTS cell viability assay

Cellular viability was assessed using a CellTiter96® Aqueous One Solution Cell Proliferation Assay (Promega, Madison, WI, USA). Briefly, mouse fibroblasts were seeded into 96-well plates (1000 cells/well in 100 μl medium). On days 1, 2, and 3, 20 μl MTS reagent was added, and the cells were incubated for 2 h. Optical density was measured at 490 nm with a Micro Plate Reader (Bio-Rad).

### RNA isolation and quantitative real-time polymerase chain reaction (rtPCR)

Total RNA was isolated from cells using the SV Total RNA Isolation System (Promega). The product was reverse-transcribed into first-strand complementary DNA (cDNA). The expression of Collagen 1 alpha 1 (Col1A1), Collagen 1 alpha 2 (Col1A2), TGFβ1, and Hsd11b1 were measured using the Power SYBR Green PCR Master Mix (Applied Biosystems, Foster City, CA, USA) according to the manufacturer's protocol. Glyceraldehyde-3-phosphate dehydrogenase (GAPDH) was used to normalize the mRNA. Sequence-specific primers were designed as follows: Col1A1, sense 5′-gagccctcgcttccgtactc-3′, antisense 5′-tgttccctactcagccgtctgt-3′; Col1A2, sense 5′- tggcccatctggtaaagaag-3′, antisense 5′- acctttgccaccttgaacac-3′; TGFβ1, sense 5′-ggagcccgaagcggacta-3′, antisense 5′-cgaatgtctgacgtattgaagaaca-3′, Hsd11b1, sense 5′-aaaattacctcctcccgatcct-3′, antisense 5′-ggcagcgagacactaccttc-3′; GAPDH, sense 5′- tgtcatcatacttggcaggtttct-3′, antisense 5′-catggccttccgtgttccta-3′. rtPCR (40 cycles of denaturation at 92°C for 15 sec and annealing at 60°C for 60 sec) was run on an ABI 7000 Prism (Applied Biosystems).

### ELISA

Blood was collected from each mouse in the evening, and the plasma corticosterone level was measured using a Cortisol EIA kit (Cayman Chemical Company, Ann Arbor, MI, USA). Skin samples were cut into small pieces, sonicated in 1% triton PBS, centrifuged for 20 min, and the supernatants were collected for analysis.

### Statistical analysis

The data are expressed as mean values ± standard deviation (SD). The Student's *t*-test was used to determine the level of significance of differences between the two groups. Analysis of variance for the groups was performed by ANOVA followed by the Bonferroni-Dunn for multiple comparisons to allow pairwise testing for significant differences between groups. Statistical significance was defined as P<0.05.

## Results

### Changes in 11β-HSD1 expression in the mouse skin with age

**Figure 1:**
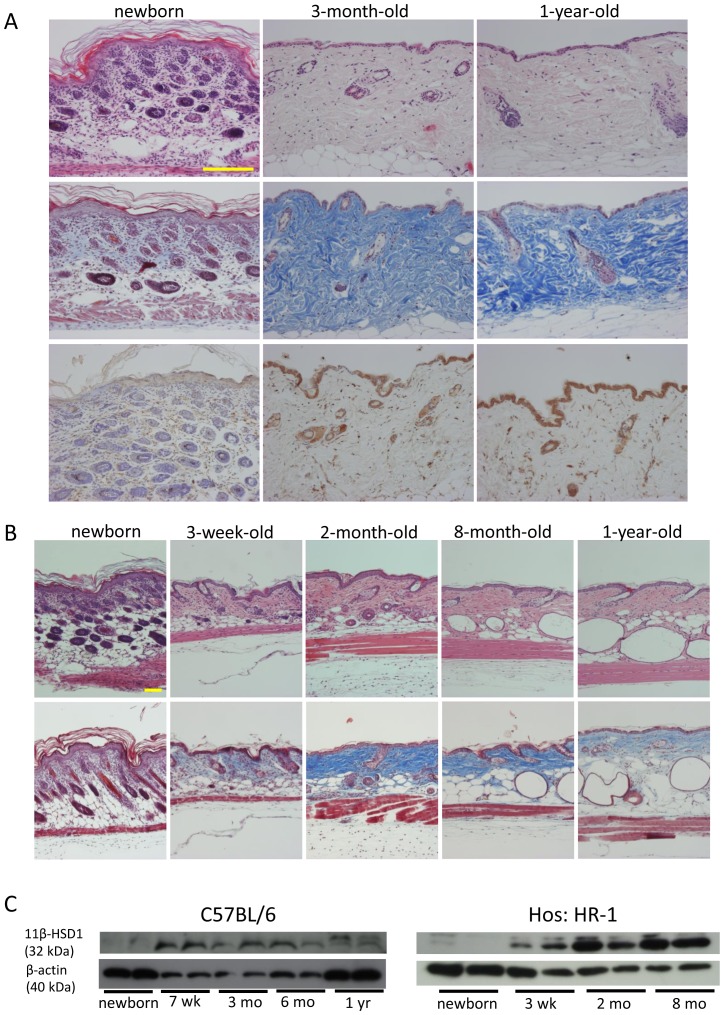
Histological analysis and changes in 11β-HSD1 expression in mouse skin during aging in C57BL/6 and Hos: HR-1 mice. (A) H&E staining (upper panel), Masson's trichrome staining (middle panel), and 11β-HSD1 staining (lower panel) of newborn, 3-month-old, and 1-year-old C57BL/6 mouse skin. Bar  = 100 μm. (B) H&E staining (upper panel) and Masson's trichrome staining (lower panel) of newborn, 3-week-old, 2-month-old, 8-month-old, and 1-year-old Hos: HR-1 mouse skin. Bar  = 100 μm. (C) Western blot analysis of 11β-HSD1 expression in mouse skin harvested from newborn, 7-weeks-old, 3-month-old, 6-month-old and 1-year-old mouse skin (C57BL/6), and newborn, 3-week-old, 2-month-old, and 8-month-old mouse skin (Hos: HR-1).

Representative H&E staining and Masson's trichrome staining of C57BL/6 mouse skin (Fig. 1A) and Hos: HR-1 (hairless) mouse skin (Fig. 1B) with age are shown. Collagen bundles in newborn mouse skin were thin and difficult to recognize with Masson's trichrome staining. In 2- to 3-month-old young mouse skin, collagen bundles increased, thickened, and were clearly visible with Masson's trichrome staining. However, in old mouse skin (1-year-old), collagen bundles decreased and became loose.

Expression of 11β-HSD1 was rarely detected in newborn mouse skin with immunohistochemistry, but was clearly detected in keratinocytes and fibroblasts of 3-month-old and 1-year-old mouse skin (Fig. 1A). The expression of 11β-HSD1 in mouse skin extract was also low in newborn mouse skin, higher in 3-week-old mouse skin, and remained high in 1-year-old mouse skin as assessed with Western blotting (Fig. 1C).

### Effects of the 11β-HSD1 inhibitor on collagen bundles and dermal thickness in young C57BL/6 mice

**Figure 2:**
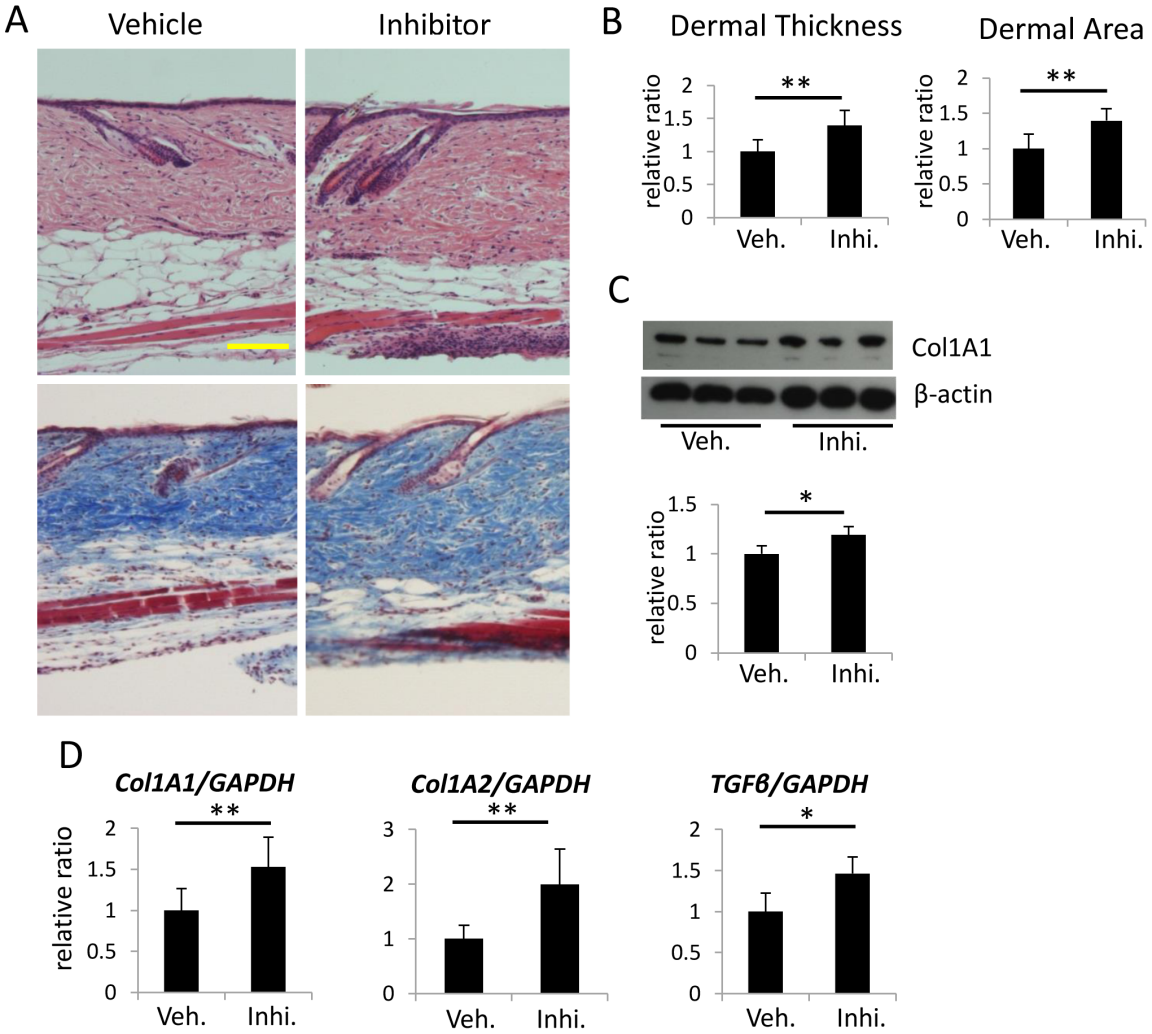
Effects of 11β-HSD1 inhibition on dermal thickness and expression of a collagen-associated gene in 7-week-old C57BL/6 mice. (A) Representative H&E and Masson's trichrome staining of 11β-HSD1 inhibitor-treated and vehicle-treated mouse skin (N = 6 per group). Bar  = 100 μm. (B) Dermal thickness and the dermal area of 11β-HSD1 inhibitor-treated and vehicle-treated mice. Dermal thickness was calculated by averaging the thickness measured at five locations in each section. Three sections from each mouse were evaluated. The dermal area in each mouse was measured using Image J. Bars show the mean epidermal thickness ± SD (N = 6; *P<0.01, Student's t-test). (C) Western blot analysis of Collagen type 1 expression in mouse skin from three individual animals treated with the 11β-HSD1 inhibitor or vehicle. Bars show the results of densitometric analysis relative to β-actin. Mean ± SD of each group are shown. (N = 3 *P<0.05, Student's t-test.) (D) The relative expression of Col1A1, Col1A2 and TGFβ1 in 11β-HSD1 inhibitor-treated and vehicle-treated mouse skin was assessed with rtPCR. GAPDH was used as an internal control. Bars indicate the mean ± SD (N = 6; *P<0.05, **P<0.01, Student's t-test).

One of the well-known side effects of topical and systemic GC is skin atrophy. We suspected that increased expression of the cortisol/corticosterone activating enzyme 11β-HSD1 in skin with age may contribute to the skin atrophy that is observed in aged mouse skin. Thus, we investigated whether subcutaneous injection of 11β-HSD1 inhibitor would affect dermal thickness and collagen production in young C57BL/6 mice. The selective 11β-HSD1 inhibitor (10 μM in 100 μl PBS) was subcutaneously injected into the right side of 7-week-old mouse back skin once a day for 21 days. DMSO dissolved in 100 μl PBS was injected as a vehicle control into the back skin on the left side. H&E staining and Masson's trichrome staining of skin sections harvested 1 day after the last injection showed increased dermal thickness in the 11β-HSD1 inhibitor-treated group compared with the vehicle-treated group (Fig. 2A, B). Furthermore, collagen type 1 expression as assessed with Western blotting in skin harvested from the 11β-HSD1 inhibitor-treated group was higher than in skin from the vehicle-treated group (Fig. 2C). Consistent with this, mRNA expressions of Col1A1, Col1A2, TGFβ1 was significantly higher in the 11β-HSD1 inhibitor-treated group compared with the vehicle-treated group (Fig. 2D).

### Effects of the 11β-HSD1 inhibitor on collagen bundles and dermal thickness in old C57BL/6 mice

**Figure 3:**
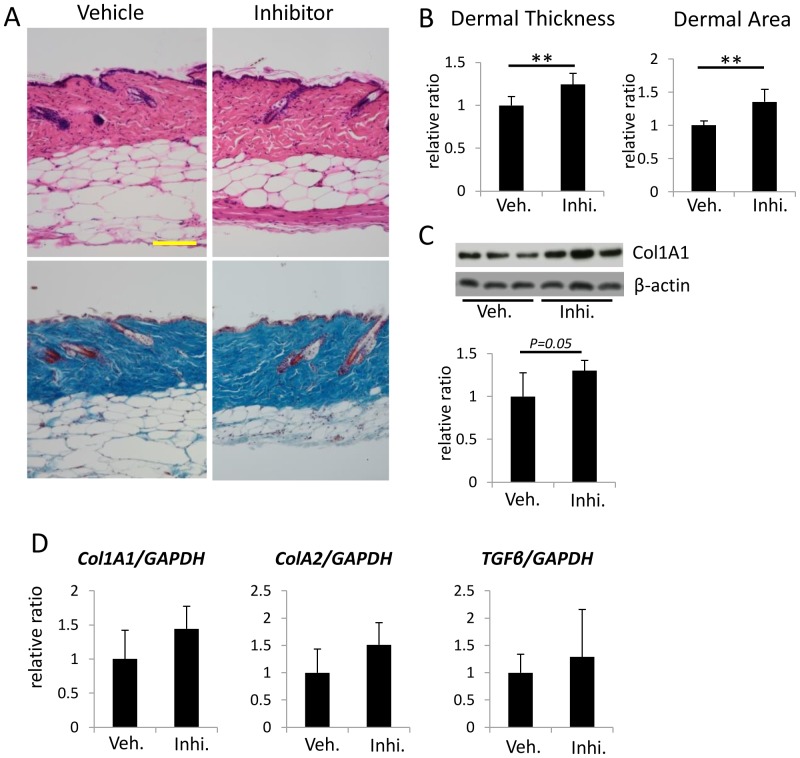
Effects of 11β-HSD1 inhibition on dermal thickness and expression of a collagen-associated gene in 1-year-old C57BL/6 mice. (A) Representative H&E and Masson's trichrome staining of 11β-HSD1 inhibitor-treated and vehicle-treated mouse skin (N = 6 per group). Bar  = 100 μm. (B) Dermal thickness and the dermal area of 11β-HSD1 inhibitor-treated and vehicle-treated mice. Dermal thickness was calculated by averaging the thickness measured at five locations in each section. Three sections from each mouse were evaluated. The dermal area in each mouse was measured using Image J. Bars show the mean epidermal thickness ± SD (N = 6; **P<0.01, Student's t-test). (C) Western blot analysis of Collagen type 1 expression in mouse skin from three individual animals treated with the 11β-HSD1 inhibitor or vehicle. Bars show the results of densitometric analysis relative to β-actin. Mean ± SD of each group are shown. (N = 6) (D) The relative expression of Col1A1, Col1A2 and TGFβ1 in 11β-HSD1 inhibitor-treated and vehicle-treated mouse skin was assessed with rtPCR. GAPDH was used as an internal control. Bars indicate the mean ± SD.

We next investigated whether subcutaneous injection of 11β-HSD1 inhibitor would affect dermal thickness and collagen production in old C57BL/6 mice. The selective 11β-HSD1 inhibitor (10 μM in 100 μl PBS) or DMSO dissolved in 100 μl PBS was subcutaneously injected into 1-year-old mouse back skin once a day for 21 days. H&E staining of skin sections harvested 1 day after the last injection showed increased dermal thickness in the 11β-HSD1 inhibitor-treated group compared with the vehicle-treated group (Fig. 3A, B). Furthermore, collagen type 1 expression as assessed with Western blotting in skin harvested from the 11β-HSD1 inhibitor-treated group was higher than in skin from the vehicle-treated group, however, they were not statistically significant (Fig. 3C). The mRNA expressions of Col1A1, Col1A2, TGFβ1 was higher in the 11β-HSD1 inhibitor-treated group compared with the vehicle-treated group, however they were not significant due to individual difference (Fig. 3D).

### Proliferation of 11β-HSD1 knockout mouse-derived dermal fibroblasts was significantly increased via the Akt pathway

**Figure 4:**
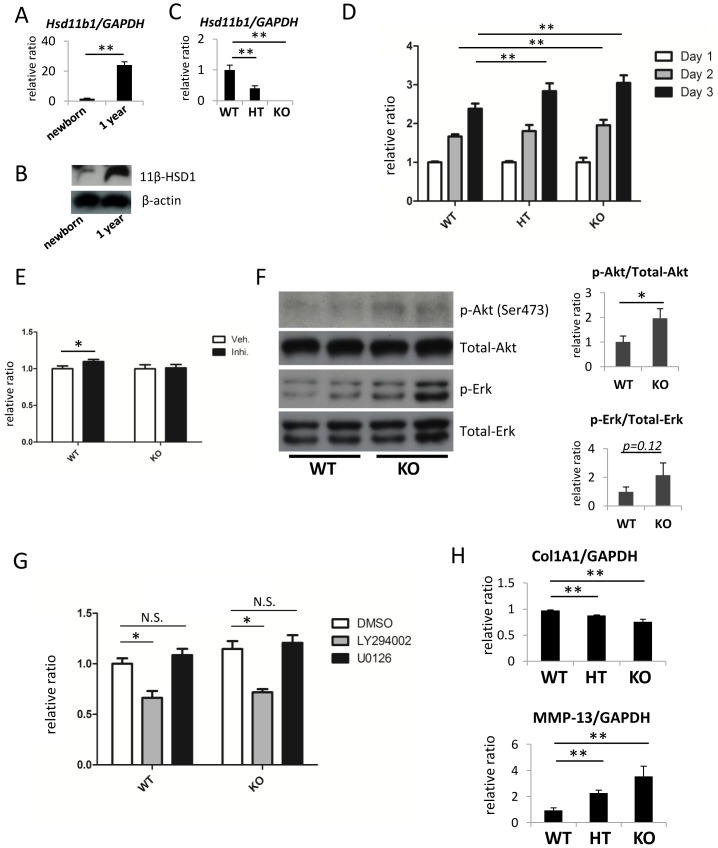
Proliferation and expression of a collagen-associated gene in 11β-HSD1 knockout mouse dermal fibroblasts. (A) The relative expression of 11β-HSD1 in dermal fibroblasts derived from newborn mouse skin and 1-year-old mouse skin was assessed with rtPCR. GAPDH was used as an internal control (N = 3; **P<0.01, Student's t-test). (B) The expression of 11β-HSD1 in dermal fibroblasts derived from newborn mouse skin and 1-year-old mouse skin was assessed with Western blotting. (C) The relative expression of 11β-HSD1 in dermal fibroblasts derived from wild-type (WT), heterozygous knockout (HT), and homozygous knockout (KO) mouse skin was assessed with rtPCR. GAPDH was used as an internal control (N = 3). **P<0.01, one-way ANOVA followed by the Bonferroni-Dunn test for multiple comparisons. (D) Proliferation of dermal fibroblasts derived from WT, HT, and KO mice was assessed with an MTS assay on days 1, 2, and 3. Day 2 and 3 data were normalized to day 1 data for each group. Bars indicate the mean ± SD [N = 6; *P<0.05, **P<0.01, two-way ANOVA followed by the Bonferroni-Dunn test for multiple comparisons. There was also a significant interaction effect of genotype and time (F = 13.33, P<0.001)]. (E) Proliferation of dermal fibroblasts derived from WT mice treated with 11β-HSD1 selective inhibitor (10 μM) for 3 days was assessed by MTS assay. DMSO was applied as vehicle control. Inhibitor group data were normalized to vehicle group. Bars indicate the mean ± SD (N = 10; *P<0.05 Student's t-test). (F) Western blot analysis of phospho-Akt (Thr308 and Ser473), Akt, phospho-Erk, and Erk in dermal fibroblasts derived from WT and KO mice. Bars show the results of densitometric analysis relative to β-actin. Mean ± SD of each group are shown. (N = 4, *P<0.05, Student's t-test) (G) The MTS assay was used to assess proliferation of dermal fibroblasts derived from WT and KO mice treated with an Akt inhibitor (LY294002, 10 μM) or an Erk inhibitor (U0126, 10 μM). Bars indicate the mean ± SD (N = 6; *P<0.0001, two-way ANOVA followed by the Bonferroni-Dunn test for multiple comparisons.) (H) The relative expression of Col1A1 and MMP-13 in dermal fibroblasts derived from WT, HT, and KO mice. GAPDH was used as an internal control (N = 3; **P<0.01, one-way ANOVA followed by the Bonferroni-Dunn test for multiple comparisons).

We next investigated the role of 11β-HSD1 in fibroblasts because the expression of 11β-HSD1 in fibroblasts derived from aged mice was significantly higher than that in newborn mice (Fig. 4A, B). Primary dermal fibroblasts were isolated from newborn skin of wild-type, *Hsd11b1^−/−^* and *Hsd11b1^+/−^* newborn mice. The expression of *Hsd11b1* was decreased in fibroblasts from *Hsd11b1^+/−^* mice and ablated in fibroblasts from *Hsd11b1^−/−^* mice (Fig. 4C). We evaluated the proliferation of these primary dermal fibroblasts using an MTS assay. Cell proliferation was mildly enhanced in fibroblasts derived from knockout and heterozygous mice, and was more obvious in knockout mouse fibroblasts (Fig. 4D). We next evaluated the proliferation of dermal fibroblasts treated with 10 μM 11β-HSD1 inhibitor. Cell proliferation was mildly enhanced in wild-type fibroblasts but not in *Hsd11b1^−/−^* mouse fibroblasts treated with 11β-HSD1 inhibitor compared with vehicle control (Fig. 4E). We further evaluated the activation of signal transduction molecules and found that phosphorylation of Akt was significantly enhanced in *Hsd11b1^−/−^* mouse fibroblasts (Fig. 4F). The Akt inhibitor, LY294002, but not the Erk inhibitor, U0126, significantly inhibited the growth of *Hsd11b1^−/−^* fibroblasts (Fig. 4G). The expression of Col1A1 was significantly decreased, and MMP-13 was significantly increased in *Hsd11b1^−/−^* - and *Hsd11b1^+/−^* mouse dermal fibroblasts (Fig. 4H).

### Evaluation of dermal thickness in *Hsd11b1^−/−^* mice

**Figure 5:**
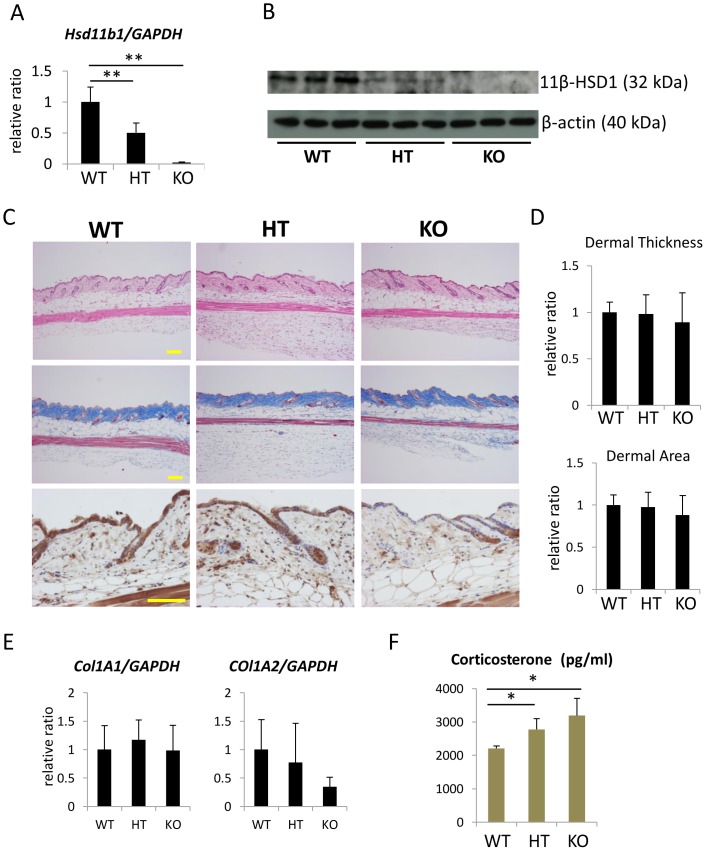
Generation of 11β-HSD1 knockout mice. (A) The relative expression of 11β-HSD1 in WT, HT, and KO mouse skin extracts was assessed with rtPCR. GAPDH was used as an internal control. **P<0.01, one-way ANOVA followed by the Bonferroni-Dunn test for multiple comparisons. (B) Western blot analysis of 11β-HSD1 in WT, HT, and KO mouse skin extracts. (C) H&E staining (upper panel), Masson's trichrome staining (middle panel), and 11β-HSD1 staining (lower panel) in 3-month-old WT, HT, and KO mouse skin. Bar  = 100 μm. (D) Dermal thickness was calculated by averaging the thickness measured at five locations in each section. Three sections from each mouse were evaluated. The dermal area in each mouse was measured using Image J. Bars show the mean epidermal thickness ± SD (N = 4). (E) The relative expression of Col1A1 and Col1A2 in 3-month-old WT, HT, and KO mouse skin. GAPDH was used as an internal control. Bars indicate the mean ± SD (N = 6). (F) Plasma corticosterone levels in WT, HT, and KO mice were measured with ELISA. Bars indicate the mean ± SD (N = 6; *P<0.05, one-way ANOVA followed by the Bonferroni-Dunn test for multiple comparisons).

Finally, we evaluated the dermal thickness in *Hsd11b1^−/−^* mouse skin. The expression of *Hsd11b1* was decreased in heterozygous mutant mouse skin and ablated in homozygous mutant mouse skin at both the protein and mRNA levels (Fig. 5 A–C). We evaluated dermal thickness and collagen expression in 3-month-old mouse skin. In contrast to the results with the 11β-HSD1 inhibitor, dermal thickness, Col1A1 and Col1A2 expressions in 3-month-old mouse skin were not different among wild-type, *Hsd11b1^−/−^* and *Hsd11b^+/−^* mouse skin (Fig. 5D, E). The level of plasma corticosterone was elevated in *Hsd11b1^−/−^* mice (Fig. 5F).

**Figure 6:**
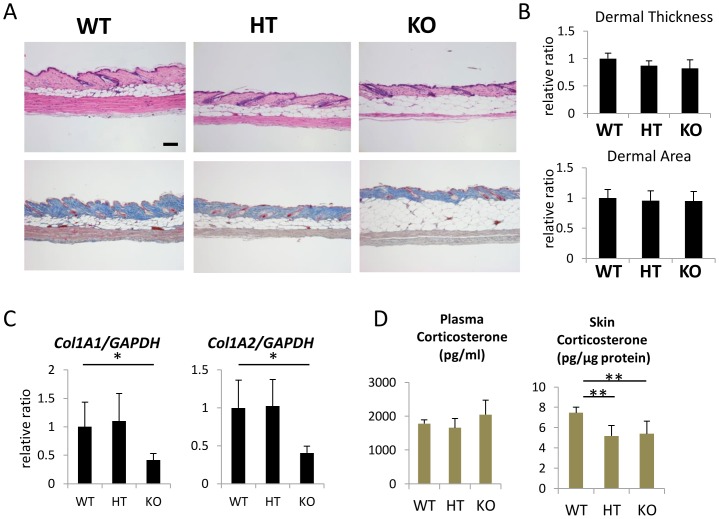
Dermal thickness and collagen type1 expression of 1-year-old 11β-HSD1 knockout mouse skin. (A) H&E staining (upper panel), Masson's trichrome staining (lower panel) of 1-year-old WT, HT, and KO mouse skin. Bar  = 100 μm. (B) Dermal thickness and the dermal area of 1-year-old mouse skin. Dermal thickness was calculated by averaging the thickness measured at five locations in each section. Three sections from each mouse were evaluated. The dermal area in each mouse was measured using Image J. Bars show the mean epidermal thickness ± SD (WT: N = 4, HT: N = 5, KO: N = 6). (C) The relative expression of Col1A1 and Col1A2 in 1-year-old WT, HT, and KO mouse skin. GAPDH was used as an internal control. Bars indicate the mean ± SD (WT: N = 4, HT: N = 5, KO: N = 6; *P<0.05, Student's t-test). (D) Plasma and skin corticosterone levels in WT, HT, and KO mice were measured with ELISA. Bars indicate the mean ± SD (WT: N = 4, HT: N = 5, KO: N = 6; **P<0.01, one-way ANOVA followed by the Bonferroni-Dunn test for multiple comparisons).

We further evaluated skin phenotypes in 1-year-old *Hsd11b1^−/−^* mice. Dermal thickness was not significantly altered in *Hsd11b1^−/−^* mice with slight increase in subcutaneous adipose tissue compared to that in wild-type mice (Fig. 6A, B). Expression of Col1A1 and Col1A2 was decreased in *Hsd11b1^−/−^* mice, contrary to the results with the 11β-HSD1 inhibitor (Fig. 6C). The level of plasma corticosterone was not different among 1-year-old wild-type, *Hsd11b1^−/−^*, and *Hsd11b^+/−^* mice. However, the skin corticosterone level was significantly lower in 1-year-old *Hsd11b1^−/−^* and *Hsd11b^+/−^* mice compared to that in wild-type mice (Fig. 6D).

## Discussion

In this study, we evaluated the role of 11β-HSD1 in dermal fibroblasts. Our data suggested that 11β-HSD1 regulates dermal collagen metabolism. We found that subcutaneous injection of a selective 11β-HSD1 inhibitor increased dermal thickness and collagen content, probably due to increased fibroblast proliferation.

Collagen content in the dermis is determined by a balance among collagen production, collagen degradation, and the number of dermal fibroblasts. Skin atrophy is a well-known and important side effect of GC treatment. GC-induced skin atrophy is reported to be associated with decreased expression of collagen type I and type III [3], [4], [34]. However, all these reports analyzed the effects of a pharmacological dose of GC on skin collagen metabolism. 11β-HSD1 is the enzyme that modulates cortisol within physiological levels [30]. Our results showed that 11β-HSD1 negatively regulates the proliferation of dermal fibroblasts, as proliferation was significantly increased in dermal fibroblasts derived from *Hsd11b1^−/−^* mice compared with wild-type mouse fibroblasts (Fig. 4B, C). In contrast to increased cell proliferation, the expression of Col1A1 was decreased, and MMP-13 was increased in *Hsd11b1^−/−^* mouse fibroblasts suggesting decreased collagen production and increased collagen degradation in *Hsd11b1^−/−^* fibroblasts (Fig. 4H). From these findings, we propose that inhibition of 11β-HSD1 increased dermal collagen content by increasing the number of dermal fibroblasts.

Epidermal and dermal atrophy, reduced numbers of fibroblasts, loss of type I procollagen expression, and increased MMP-1, 3, and 9 expression are observed in intrinsically aged human skin [35], [36], [37], [38], [39]. Decreases in collagen content were also observed in 1-year-old mouse skin compared with 3-month-old mouse skin. We propose that local cortisol activation by 11β-HSD1 may be involved in dermal atrophy in intrinsic aging, as the expression of 11β-HSD1 increased with age in mouse skin. We also found in this study that 11β-HSD1 was a negative regulator of fibroblast proliferation.

To further clarify the role of 11β-HSD1 *in vivo*, we evaluated the skin from 3-month- and 1-year-old *Hsd11b1^−/−^* mice. In contrast to our findings using the 11β-HSD1 inhibitor, the dermal thickness of 1-year-old *Hsd11b1^−/−^* mice was not affected, and significant decreases in Col1A1 and Col1A2 expression were observed (Fig. 6). The action of cortisol/corticosterone is determined by both local and systemic cortisol/corticosterone. In 1-year-old *Hsd11b1^−/−^* mice, the level of skin tissue corticosterone was decreased. However, plasma corticosterone was significantly increased in 3-month-old mice and tended to increase (P = 0.09) in 1-year-old *Hsd11b1^−/−^*. Dermal atrophy is induced by long-term exposure to cortisol/corticosterone through life. Regarding the discrepancy between local and systemic corticosterone in *Hsd11b1^−/−^* mice, clarification of the role of 11β-HSD1 in dermal collagen metabolism in this mouse is difficult. Increased plasma corticosterone levels were also reported in previously generated *Hsd11b1^−/−^* mice, and the authors discussed that increased plasma cortisol levels may be the result of an absence of negative feedback in the central nervous system due to an absence of 11β-HSD1 activity in the central nervous system [41]. Improved collagen density and rescued procollagen assembly-associated gene expression have been reported recently in 2-year-old *Hsd11b1^−/−^* mice [40]. Decreased local corticosterone may overcome the increased systemic corticosterone in 2-year-old mice.

We hypothesize that increased subcutaneous adipose tissue in *Hsd11b1^−/−^* mice may also affect dermal structures. Ezure and Amano reported the importance of subcutaneous adipose tissue in dermal structures [42]. In the dorsal skin of mice fed a high-fat diet, the dermal layer became significantly thin due to a decreased number of dermal fibroblasts, and this was accompanied by a significantly thickened subcutaneous adipose tissue layer. However, the dermal thickness of ear pinna, which does not contain subcutaneous adipose tissue, was not changed [42]. Increased subcutaneous adipose tissue might also affect dermal structure in *Hsd11b1^−/−^* mice.

In summary, we found that 11β-HSD1 regulated collagen metabolism in mouse skin. We also found that the expression of 11β-HSD1 increased with age in mouse skin. An 11β-HSD1 inhibitor may have the potential to reverse the decreased collagen content that is observed in intrinsically and extrinsically aged skin and in the skin atrophy that is induced by GC treatment.
